# Notes and News

**Published:** 1893-02-18

**Authors:** 


### Notes and News.

Collected and Communicated.

"Out of sight out of mind" appears too often be to the fate of institutions as well as of individuals, and it is pleasant to be able to record an experience of a different character. The North-Eastern Hospital for Children has recently received a donation of £700 from Baron Hirsch—and a legacy of £500 is also announced. The North-Eastern is situate in one of the most densely populated districts of North-east London. It has suffered very severely from the neglect of the charitable public, and the executive have had a sharp struggle to maintain the hospital on its proper footing. It may be hoped that they have now turned the corner. The festival dinner will be held on May 9th next.

The London Vegetarian Society, in Farringdon Street, state that they are prepared to give specimen halfpenny dinners in schools gratis. They propose to demonstrate the possibility of providing self-supporting, nourishing dinners for the sum of a halfpenny per head. We notice one of the chief items on the programme is wholemeal bread. No more excellent form of food for children exists than really well made wholemeal bread. Unfortunately, as a rule, the liking for brown breads is an acquired taste, and therefore the staple food of the poorer children is not as nutritive as it might be were the use of whole meal bread more general. This bread contains all the constituents necessary to nourish and sustain life, a quality not shared by any other article of food, and if the liking for such bread were but fostered by teachers and those in whose power it is to influence both parents and children, a healthier and better nourished race would be the result. The objections to brown breads are removed where they are properly manufactured. We ourselves, as the result of a wide experience, feel that no one has succeeded better in the production of a perfect bread than Messrs. Meaby, of Reading. Their "Triticumina" bread we consider unrivalled, and we are supported in our opinion, by good authorities on the subject. The choice of such a staple commodity of children's diet is of the utmost importance, and we therefore feel justified in laying so much stress on the choice of a really good bread.

The City of London Truss Society held its annual meeting on the 1st inst. The report stated that last year the number of instruments supplied to patients was 9,652, and the income, inclusive of legacies, was unusually large. The demands on this charity continue to increase, and the alterations and enlargement of the premises in Wilson Street recently decided upon are being rapidly proceeded with, and these, when complete, will materially add to the comfort of the patients—especially the female patients. The annual festival will be held on May 16th next, and we sincerely hope that the society will then receive the generous support which it so well deserves.

We have joined in the protest against "penny dreadfuls" with their pernicious effect more especially on the young of the lower classes. There are already numbers of rival penny productions of a much better class which would be equally popular and, at the same time harmless, which should be better known. The young are very precocious nowadays, and they soon demand "grown up" stories. We are glad to welcome the endeavour of Messrs. Howe, of Paternoster Row, to place wholesome literature within the reach of all. They have commenced a series of penny numbers, entitled "Everybody's Stories, Old and New." No. 1 of Dickens's "Christmas Carol" and No. 2 is also devoted to an "old" story. We trust that in the choice of newer literature Messrs. Howe will keep up high standard in these productions, and they will be doing a good service to many.

It is frequently said that women are lacking in enterprise. But that is fast becoming an "old world" theory, as it cannot in justice be held true in the "new world." In America women try their hand at business of almost every description, and are mostly quite successful in their undertakings. There is a good lady in Newburgh who is stated to have carried on the street cleaning and sprinkling business with profit and success during seventeen years. Her staff consists of twenty men, but she does her own collecting, and herself superintends the work. It may not be generally supposed that the profits from street cleaning are large, but the last report of the Commissioners of Sewers shows the value of waste products in a new light. A revenue of £1,133 has been derived from this source during the past year. Bottles, boots, knives, iron, corks, string, paper, rags, and metals formed the marketable produce of the rubbish of the City streets.

There should be a great awakening throughout the country in respect to the why and wherefore in practical common-sense matters. The County Councils are encouraging technical education everywhere, and technical institutes are new departures in the realm of architecture. These technical schemes include hygiene, nursing, cooking, and dairy work to a largely increasing extent. So great is the demand for this class of instruction that there is a dearth of really competent teachers, especially in nursing and hygiene, and as in some cases the teachers have had to learn alongside of the taught, some rather quaint incidents have occasionally resulted from imperfect acquaintance of the subject by the lecturer. A scheme has been started to supply really well-trained ladies as teachers in the two branches named above, which was set forth in our issue of Nov. 19th. This offers an excellent field of employment for ladies without a great preliminary outlay, and we expect that many desirous of a useful occupation will benefit themselves and others by taking up the work, of which particulars can be had by writing to Miss Lamport, 55, Burton Crescent, W.C.

